# A First Principles Study of Lithium Adsorption in Nanoporous Graphene

**DOI:** 10.3390/nano14181528

**Published:** 2024-09-20

**Authors:** Liudmyla Barabanova, Alper Buldum

**Affiliations:** 1 Department of Chemistry, The University of Akron, Akron, OH 44325, USA; lvb5@uakron.edu; 2 Department of Mechanical Engineering, The University of Akron, Akron, OH 44325, USA

**Keywords:** nanoporous graphene, lithium adsorption, ab initio calculations, lithium-ion batteries

## Abstract

Recently, nanoporous graphene has attracted great interest in the scientific community. It possesses nano-sized holes; thus, it has a highly accessible surface area for lithium adsorption for energy storage applications. Defective graphene has been extensively studied. However, the lithium adsorption mechanism of nanoporous graphene is not clearly understood yet. Here, we present theoretical investigations on the lithium-ion adsorption mechanism in nanoporous graphene. We perform ab initio electronic structure calculations based on density functional theory. Lithium adsorption in a graphene nanopore is studied and adsorption sites are determined. We also study different lithium-ion distributions in graphene nanopores to determine the best lithium–nanoporous graphene structures for lithium-ion batteries. We show that lithium ions can be adsorbed in a graphene nanopore, even in a single layer of graphene. It is also shown that adding more nanopores to multilayer nanoporous graphene can result in higher Li storage capacity for new-generation lithium-ion batteries.

## 1. Introduction

Lithium-ion batteries (LIBs) have developed tremendous popularity in recent years as promising renewable energy storage systems. Their high capacity, relatively low cost, and high power and energy density make LIBs very desirable for electric vehicles and energy storage applications [1,2,3]. However, lower lithium density in batteries is a problem in battery production [4]. In LIB production, a commonly used anode material is graphite oxide. However, it was not the answer, considering the increasing storage capacity demands [5,6,7]. In search of a better anode material to increase the storage capacity, LIB manufacturers recently considered graphene-based and other carbon materials as promising materials for energy storage in LIBs and supercapacitors [5,6,7,8,9]. 

Graphene, a 2D sheet of sp^2^-hybridized carbon, has superior chemical and physical properties [10,11,12]. It has attracted great interest in energy storage and conversion [13], catalysis [14,15], gas adsorption and separation [16], biomedical [17], and environment [18] applications. Due to these properties, graphene has been considered as one of the most promising materials to replace graphite in next-generation lithium-ion batteries (LIBs) [19]. 

Initially, it was believed that Li would be adsorbed on both sides of graphene [20], forming Li_2_C_6_ stoichiometry (with a theoretical specific capacity of 744 mAh g^−1^). Thus, it would contain twice the number of lithium ions compared to stage-one intercalated graphite. It appears that there can be a strong Coulomb repulsion between the Li ions, resulting in much less Li storage density [21,22]. Recently, morphologically engineered graphene anodes with defective, porous, spheric, holey, or nanoribboned structures with much higher storage capacities were reported [19]. Furthermore, graphene-derivative anodes with heteroatom doping and covalent functionalization with high-storage capacities were also reported. An extensive list of these graphene-based anodes and their reported storage capacities can be found in reference [19]. In the list, some of the most promising graphene-based anodes are porous graphene, defective graphene, and holey graphene with capacities up to 930 and 1040 mAh g^−1^. On the other hand, larger active lithium loss in the first several cycles results in less initial Coulombic efficiency, which restricts practical applications [19]. Another challenge is the large-scale production of the high-quality graphene with the desired morphology. 

Density functional theory (DFT) is a very valuable instrument or tool for the investigations of materials properties [23,24]. It became very important for the understanding and advancement of the mechanical, chemical, and electronic properties of graphene [25,26]. Nakada and Ishii performed DFT calculations of adsorption and migrations of various atomic species on graphene for the formation of nanostructures on graphene [27]. DFT even provides valuable insights for the low-temperature CVD growth of graphene. Recently, O. Tau et al. identified most probable Cu surface-catalyzed reactions during the low-temperature CVD growth using aromatic hydrocarbon precursors [28]. 

There are various theoretical investigations of graphene or graphene-based materials for lithium-ion-battery anodes. Lee and Persson studied Li adsorption and intercalation in single-layer graphene and few-layer graphene by performing density functional theory (DFT) calculations [22]. They considered metallic Li atom energy as a base energy, which is the cohesive energy of metallic lithium. They found that there exists no Li arrangement that stabilizes Li adsorption on the surface of a single-layer graphene unless there are defects in the graphene. 

Buldum and Tetiker showed that one of the most promising graphene–lithium combined structures contain a single graphene layer and four lithium layers (two Li layers on each side of graphene) [29]. They also found that single-layer graphene–Li structures are the worst due to the Coulomb repulsion between the positive Li ions as there is not much electron density between the Li ions to screen the Coulomb repulsion. This is consistent with the previous theoretical investigations. Meanwhile, with the addition of extra Li layers on both sides, the Coulomb repulsion between the Li ions is screened more effectively due to the electron charge density between the Li ions. The adsorption energy per lithium in this structure (1.73 eV) was slightly above the cohesive energy of metallic lithium (1.68 eV). 

Y. Liu et. al. performed DFT calculations of interactions of Li with a wide variety of sp^2^ C substrates including pristine, defective, and strained graphene [30]. They also calculated the work required to fill the unoccupied electronic states of the substrates. D. Datta et al. investigated enhanced lithiation of defective graphene with divacancy (DV) and Stone–Wales (SW) defects [31]. They stated that Li storage capacities up to 1675 mAh/g can be achieved. Y.J. Tsai and C.L. Kuo performed first-principles calculations to study the effects of structural disorder in the graphene substrate on Li storage capacity [32]. They found that Li storage capacity is largely determined by the local geometry of the defect sites. Many of these studies were on defective graphene. However, the lithium adsorption mechanism of nanoporous graphene is not clearly understood yet.

Defective graphene has been extensively studied. We believe there are no such theoretical investigations on the adsorption mechanism of lithium in nanoporous graphene in the literature. Here, we present theoretical investigations on the lithium-ion-adsorption mechanism in a graphene nanopore. Ab initio electronic structure calculations were performed based on density functional theory. We also studied different lithium-ion distributions in graphene nanopores to determine the best lithium–nanoporous graphene structures for LIBs. We show that lithium ions can be adsorbed in a graphene nanopore, even in a single layer of graphene. We also show that adding more nanopores to multilayer nanoporous graphene can result in higher Li storage capacity. These findings can be very valuable for the fundamental understanding of lithium adsorption in graphene-based materials and for energy storage applications. 

The methods used in our calculations are presented in Section 2. In Section 3, our results are presented. Lithium-ion transport through the nanopores for different pore sizes is presented in Section 3.1. Lithium-ion adsorption in a graphene nanopore is studied by investigating the effect of the graphene pore size, lithium-ion placement, and the lithium height above the graphene surface, which is presented in Section 3.2. Many lithium ions were distributed in graphene pores, and they were evaluated based on the adsorption energy per lithium atom, the adsorption energy per carbon atom, and lithium density. Investigations of multilayer lithium–nanoporous graphene structures and their evaluation for lithium-ion battery anodes are in Section 3.3. Our conclusions are given in Section 4.

## 2. Methods

Our electronic structure and geometry optimization calculations are based on density functional theory (DFT). The Quickstep module [33] in the CP2K software package v9.1 was used. Periodic structures can be investigated using this module. The exchange–correlation term in the Kohn–Shams equations was approximated using the Perdew–Burke–Erznerhof (PBE) [34] functional equation with generalized gradient approximation (GGA). The DZVP-MOLOPT-SR-GTH [35] basis set and the Goedecker–Teter–Hutter (GTH) [36] pseudopotential were used with a cut-off energy of 900 Ry for the density grid. The Van der Waals interactions were included using the semi-empirical ‘DFT+D3’ term [37]. The LBFGS [38] optimizer was used for energy minimization to optimize the geometry.

## 3. Results 

### 3.1. Lithium Ion Transport through Graphene Nanopores 

**Figure 1:**
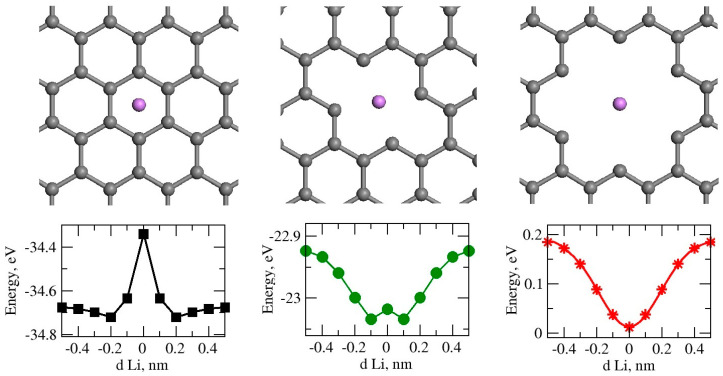
Top views of the pore structures and variation in total energy of as a function of Li-graphene pore separation d_Li_ perpendicular to graphene plane for C0H, C2H and C6H graphene pore sizes are presented. Periodic boundary conditions are used in all three directions. There is a 30-angstrom gap in the perpendicular direction.

First, we studied lithium transport through graphene nanopores. An atomic structure model of a single layer of pristine graphene was created. The model contained 540 atoms with supercell sizes of 36.89 × 38.34 × 30.00 Å. Periodic boundary conditions were used in all three directions. There was a 30.00 Å gap between the graphene sheet and its image in the z-direction. By removing carbon atoms from the pristine graphene, pores of different sizes were created. The atoms were removed from the center of the pristine graphene. More details about the models are provided in the Supplementary Materials. The model structures labelled as C0H, C2H, and C6H, with 0, 2, or 6 carbon atoms removed from the graphene atomic structure, are presented in Figure 1. The lithium atom was placed 0.5 nm above the graphene plane, and it was translated towards the center of the pore in discrete steps. Ab initio total energy calculation was performed in each step. No potential energy barrier was observed when the lithium atom passed through the center in the C6H model. Therefore, we selected model C6H for part of the further studies.

### 3.2. Adsorption Mechanism of Lithium in a Graphene Nanopore

In addition to the model C6H, two new nanopore models (C24H and C54H) were created with 24 and 54 removed carbon atoms from the center of the graphene structure. The pore diameters in the C24H and C54H models are 14.36 and 17.04 Å, respectively. In these structures, the pore areas are 1.62 nm^2^ and 2.28 nm^2^. The pristine graphene area was 14.14 nm^2^. Thus, the pore area/pristine graphene area ratios are 0.11 for C24H and 0.16 for C54H. Please note that these ratios depend on the selection of graphene model created.

**Figure 2:**
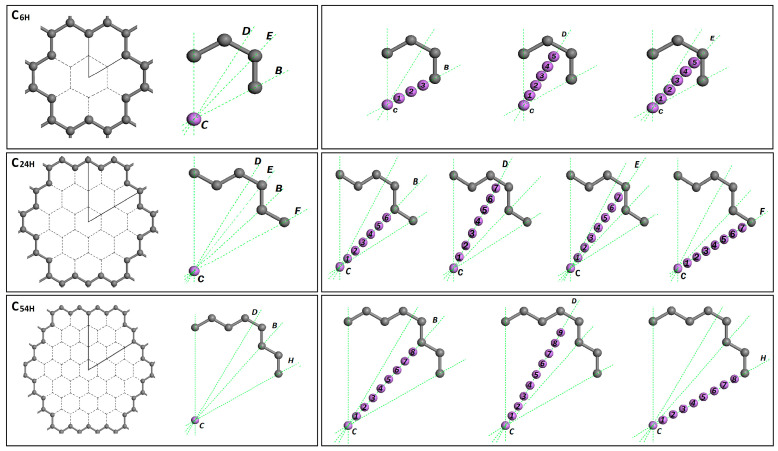
The graphene pore symmetrical parts and important translation lines or directions for models C6H, C24H, and C54H.

Using these three model structures, we investigated the lithium adsorption in a graphene nanopore. For all these three models, the symmetry of the graphene pore structure was studied and important translation lines or directions were identified. As can be seen in Figure 2, these important translation lines start from the center (C) site of the graphene pore and end at the pore edge. In the figure, B-, E-, F-, and H-lines end at carbon atoms, and the D-line ends at the carbon–carbon (C-C) bond.

Lithium ions were placed and translated along these lines, and preferred adsorption sites were identified. The variation in total energy was calculated for each line. During these calculations, the lithium ion was placed at a height of 0.0 nm or 0.172 nm above the graphene pore surface. We found that 0.172 nm was the optimized height of a lithium ion on the pristine graphene.

**Figure 3:**
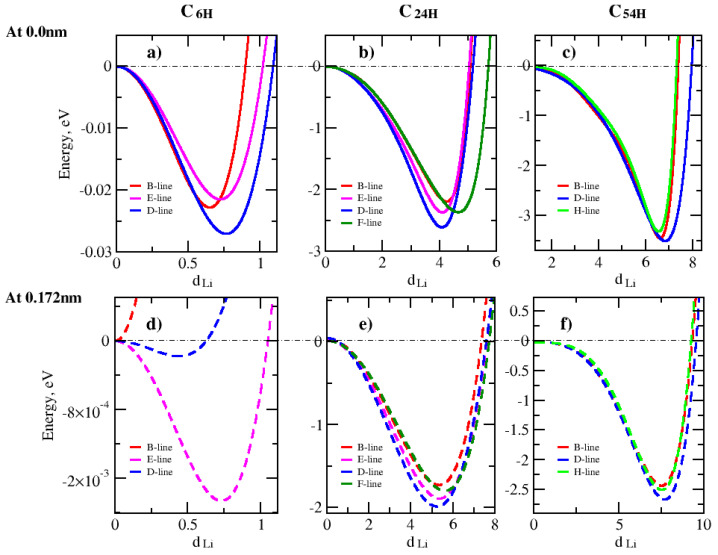
Total energy of the structure as a function of Li-graphene pore edge separation d_Li_ parallel to graphene plane for C6H, C24H, and C54H graphene pore sizes. The energy values are relative to the C-site energy in each model’s structure. (**a**–**c**) are for zero lithium ion height for C6H, C24H and C54H, respectively. (**d**–**f**) are for 0.172 nm lithium ion height with respect to the graphene plane for C6H, C24H and C54H, respectively.

Lithium-ion adsorption in a graphene nanopore is studied by investigating the effect of the graphene pore size, lithium-ion placement, and the lithium height above the graphene surface. First, we investigated the effect of graphene pore size on the lithium adsorption. Figure 3 presents the variation in total energy along the Li-ion translation lines for different pore sizes.

To determine the lowest energy positions for the lithium adsorption, various translation directions or lines were selected inside the graphene pores as they can be seen in Figure 2. The B-, E-, F-, and H-lines end at carbon atoms, and the D-line ends at the carbon–carbon (C-C) bond. By comparing the results in Figure 3, it appeared that the directionality of lithium ions’ placement inside graphene pores has a direct impact on the lithium adsorption mechanism. This study shows that the lowest energy values are along the D-line in the C6H, C24H, and C54H structures at distances from the center (d_Li_) = 0.75, 4, and 7 angstroms, respectively. The lithium height is 0.0 nm.

**Figure 4:**
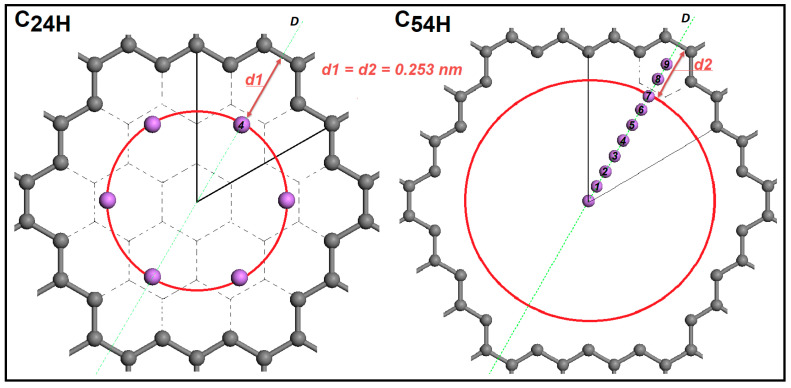
Li-graphene pore edge distance for C24H and C54H pore sizes along D-line with the lowest energy positions presented for C24H.

Another interesting result from these investigations is that the lowest energy position of the Li-ion relative to the center of the C-C bond is the same for different pore sizes. It is 0.253 nm away from the C-C bond in both C24H and C54H models. In Figure 4, d_1_ and d_2_ distances represent these distances, and they are equal. This information gives us the opportunity to place multiple Li-ions in the pore. On the other hand, lithium-ion–ion repulsion may limit the number of ions placed in the pore.

**Figure 5:**
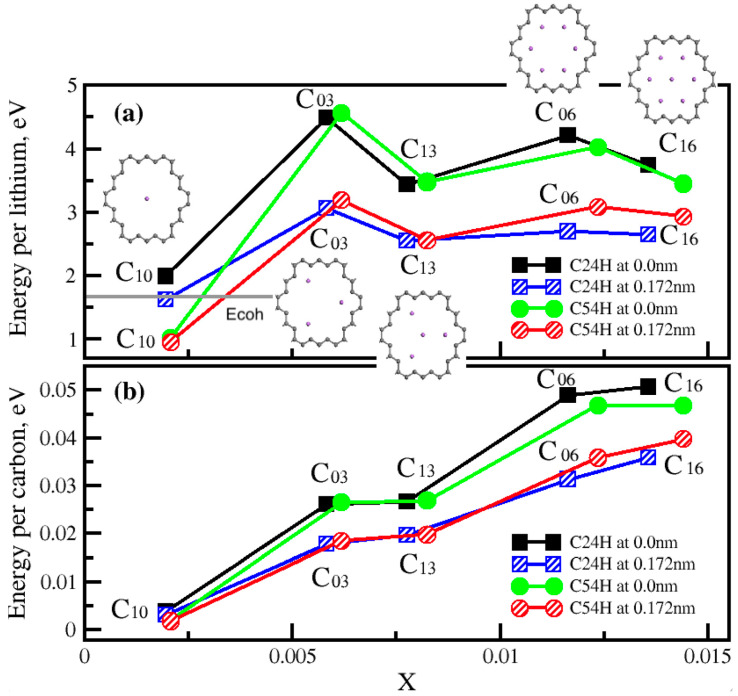
Adsorption energy per lithium (**a**) and adsorption energy per carbon (**b**) as a function of lithium density, x, of single-layer nanoporous graphene with Li assembly inside the pore at 0.00 nm and 0.172 nm heights. The cohesive energy of metallic lithium is represented by a grey line in (**a**). E_coh_ = 1.68 eV.

The lithium-ion distribution inside the C24H and C54H pores and their adsorption energies were studied. New models from C24H (C10, C03, C13, C06 and C16) were created and the D-lines were used to distribute the lithium ions. Figure 5 presents the models for the C24H pore size. In the same way, we also created the new models for C54H.

The adsorption energies per lithium and per carbon atoms were determined next. The total adsorption energy is presented as follows:

*E* = −(*E_GLi_* − *N_Li_* × *E_Li_* − *E_G_*) (1)

where *E_GLi_* is the total energy of the combined structure, *N_Li_* is the total number of lithium ions in the structure, *E_Li_* is the total energy of an isolated lithium ion, and *E_G_* is the total energy of an isolated mono- or bilayer graphene. In some of the theoretical investigations, the cohesive energy of metallic lithium is used for *E_Li_*. We also consider the cohesive energy of metallic lithium, *Ecoh* = 1.68 eV [39] and compare adsorption energy per Li atom in graphene nanopores with this value.

In Figure 5, adsorption energy per lithium and adsorption energy per carbon are presented. They are functions of lithium density, x, per carbon. Adsorption energy per carbon increases, but energy per lithium saturates when we increase the lithium-ion density, and it does not decrease as long as we do not place a lithium ion at the center. Other lithium ions are symmetrically distributed facing the C-C bonds in the best lithium distribution model structures. The cohesive energy of metallic lithium is presented as a grey line in Figure 5a. We found that energy per Li is higher than *Ecoh* in most cases; thus, these nanopores can contain multiple Li ions. C03 and C06 appeared to be the best lithium–nanoporous graphene structures in a single-layer graphene.

### 3.3. Multilayer Lithium–Nanoporous Graphene Structures

**Figure 6:**
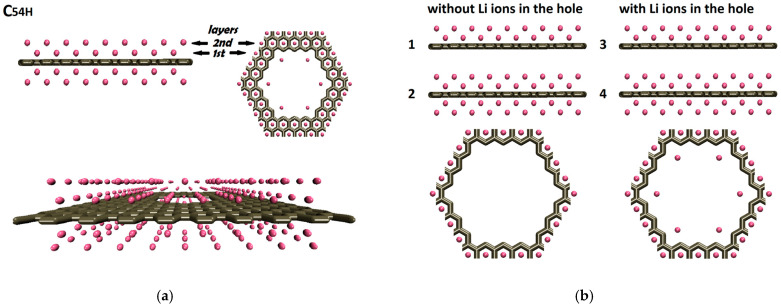
(**a**) Multilayer lithium–nanoporous graphene structures. (**b**) Side views of the atomic structures of the models M1–M4 numbered 1 through 4 and top views of the pores.

After determining the best lithium distribution inside the graphene pore, we focused on creating lithium–nanoporous graphene combined structures. Ten new model structure models were created. Here, the idea was to optimize the lithium–nanoporous graphene combined structure for the highest lithium-ion density. The first four models are shown in Figure 6.

The C54H pore was used to create these model structures. Buldum and Tetiker performed a systematic study of single- and multilayer graphene–lithium combined structures [29]. Various atomic graphene–lithium-model structures were relaxed and investigated. Our model structures here were based on these already relaxed structures, and static total energy calculations were performed. In the C54H model, the first lithium layer–graphene separation was 0.184 nm, and the second lithium layer was placed 0.203 nm above the first lithium layer, with respect to the data obtained from reference [29]. No lithium ions were distributed above and below the graphene pores (Figure 6a). These four models were divided into two categories: without Li ions inside the pore (models M1, M2) and with Li ions inside the pore (models M3, M4 which are based on C06 lithium assembly at 0.0 nm). Models M1 and M3 only had two lithium layers above the nanoporous graphene surface. Models M2 and M4 had an additional two layers of lithium at the bottom of the nanoporous graphene (Figure 6b).

**Figure 7:**
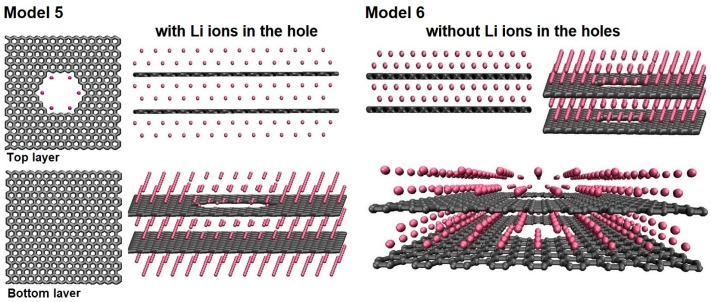
The atomic structures of models M5 and M6 with and without Li inside the pores, respectively.

The next two models created (M5 and M6) contained bilayer graphene structures. The combination of a nanoporous graphene and a pristine graphene was performed in model M5. A bilayer containing two identical nanoporous graphene layers in model M6 is presented in Figure 7. Two additional lithium layers around the second pristine graphene layer were added in model M5, while model M6 still had four lithium layers distributed around the first nanoporous graphene layer.

Total energy calculations were performed to determine the correct separation distance between the graphene layers. For both structures, the lowest energy was observed at a separation distance of around 0.6 nm between the graphene layers, and the distance between the bottom graphene and the lithium layer above it was 0.213 nm.

**Figure 8:**
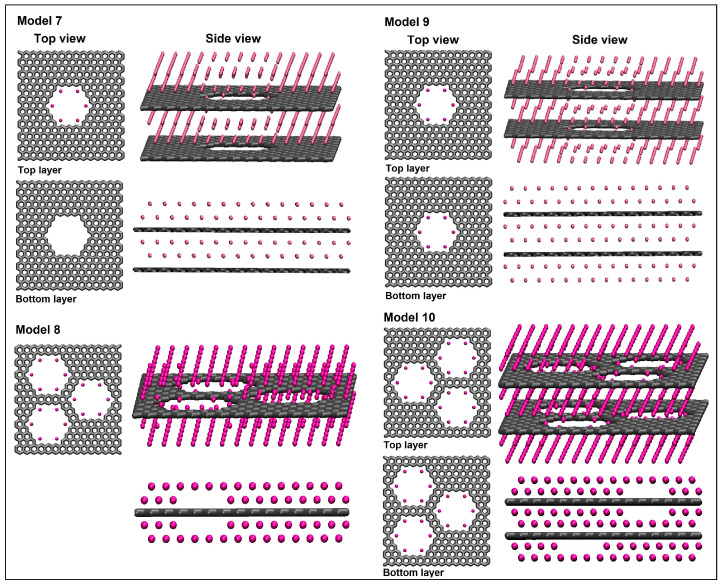
The structures for models M7–M10.

Models (M7–M10) are presented in Figure 8. Like M6, the lowest energy for models M7, M9, and M10 was found at the separation of around 0.6 nm between graphene layers. Model M7 is like model M6; however, six lithium ions were distributed inside the top graphene pore. In model M9, we added another six lithium ions inside the bottom graphene pore and an additional two lithium layers below the second graphene sheet. In models M8 and M10, the number of pores in a single graphene layer was increased to three. Model M8 contains a single layer of nanoporous graphene with two lithium layers above and below it, with additional lithium ions assembled inside all three pores. Model M10 contains a bilayer of nanoporous graphene with three pores in each graphene layer.

**Figure 9:**
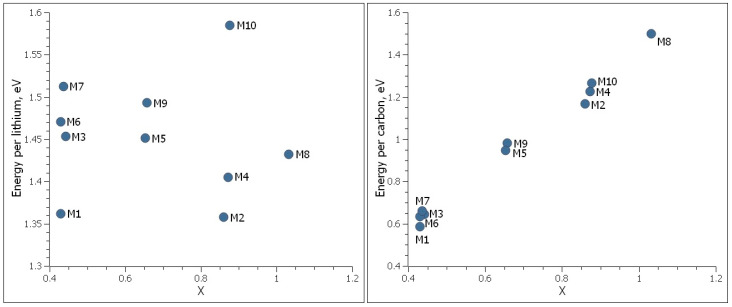
Adsorption energy per lithium and adsorption energy per carbon as a function of lithium density, *x*, of single- and bilayer nanoporous graphene structures. The cohesive energy of metallic lithium is E_coh_ = 1.68 eV.

The adsorption energy per lithium and adsorption energy per carbon were calculated for these models and are shown in Figure 9. We found that Model M10 has a better atomic structure than all other models, due to its highest energy per lithium with high lithium density. On the other hand, its energy per lithium is still lower than the cohesive energy of metallic lithium. The next best structures are M7 and M9. Since M7 has two lithium layers missing at the bottom of the second nanoporous graphene sheet, it has a lower lithium density in comparison to M9. Model M8 has high lithium-ion density; however, it has significantly less adsorption energy per lithium, as it contains only a single layer of nanoporous graphene.

These investigations show that structures containing Li in the pores and in between the graphene sheets have higher adsorption energy per lithium. Multilayer nanoporous graphene with pores are found to be more favorable. There are no favorable single-layer nanoporous graphene structures if they do not contain many nanopores. Lithium ions will likely be only in the nanopores of single-layer nanoporous graphene structures. Adding nanopores to single-layer graphene may help to increase the Li capacity. Bilayer nanoporous graphene will contain more lithium due to intercalation between the layers. We are not aware of any experimental study of the direct comparison of the results; however, the porous graphene network structures designed by Z. Fan et al. have slightly larger nanopores (3–8 nm pore size) and they report a high reversible capacity of 1723 mAh g^−1^ [40]. We believe, the best structures will be multilayer nanoporous raphene structures and adding more nanopores to multilayer nanoporous graphene can result in higher Li storage capacity.

## 4. Conclusions

Nanoporous graphene-based anode materials possess nano-sized holes, and they have highly-accessible surface areas for lithium adsorption for energy storage applications. There are many studies on defective graphene; however, the lithium adsorption mechanism of nanoporous graphene is not clearly understood yet. We performed theoretical investigations on the lithium-ion-adsorption mechanism in nanoporous graphene. Different lithium-ion distributions in graphene nanopores were also created and studied to determine the best lithium–nanoporous graphene structures for lithium-ion batteries. The lithium ions preferred particular adsorption sites, which were close to the center of the C-C bonds at the edge of the pore. More lithium ions were distributed in the pores considering these preferred adsorption sites. These lithium-ion distributions in graphene nanopores were evaluated. We found that lithium ions can be adsorbed in a graphene nanopore even in a single layer of graphene without forming metallic lithium clusters or dendritic structures. Thus, lithium ions will likely be only in the nanopores of single-layer nanoporous graphene. On the other hand, adding more nanopores to the nanoporous graphene can result in higher Li storage capacity. Final part of this study was the creation of multilayer lithium–nanoporous graphene-model structures and their evaluations. Our studies show that structures containing Li in the pores and in between the graphene sheets have higher adsorption energy per lithium atom. Multilayer nanoporous graphene with increased number of pores is one of the most promising materials for energy storage applications.

## Data Availability

Data is contained within the article or Supplementary Materials.

## Supplementary Materials

The following supporting information can be downloaded at: https://www.mdpi.com/article/10.3390/nano14181528/s1, File S1: Supplementary information.
