# Supplementary material for: A First Principles Study of Lithium Adsorption in Nanoporous Graphene
Source: Nanomaterials (Basel). 2024 Sep 20;14(18):1528. doi: 10.3390/nano14181528 (PMC11435369; doi:10.3390/nano14181528)
Supplement: Supplementary file 1 [file nanomaterials-14-01528-s001.zip › nanomaterials-3207925-supplementary.pdf]

SUPPLEMENTARY INFORMATION

In the manuscript the nanoporous graphene structures used in figures 1 to 5 are based on the single layer of pristine graphene nanosheet. The pristine graphene supercell was created with the following parameters:

(a). **Pristine graphene model C0H (0 carbon atoms removed) with lithium atom at the center of the hexagon ring** (related with figure 1 in the manuscript):

- number of carbon atoms: 540
- number of lithium atoms: 1
- cell vectors A x B x C: 36.8927 x 38.3400 x 30.000Å
- graphene unit cell with a_x = 2.46Å, a_y = 4.26Å and C-C bond distance 1.42Å

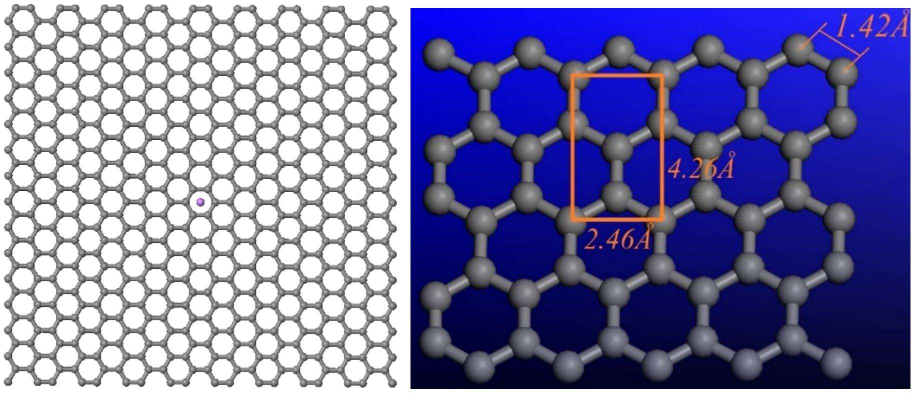

(b) **Graphene nanopore model C2H (2 carbon atoms removed) with lithium atom at the center of the pore**: the C2H nanopore was created by removing 2 carbon atoms from the pristine graphene model described above in (a)

- number of carbon atoms: 538
- number of lithium atoms: 1
- cell vectors A x B x C: 36.8927 x 38.3400 x 30.000Å

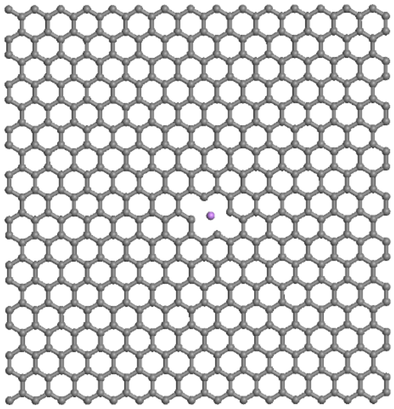

(c) **Graphene nanopore model C6H (6 carbon atoms removed) with lithium atom at the center of the pore**: the C6H nanopore was created by removing carbon atoms from the pristine graphene model described above in (a)

- number of carbon atoms: 534

- number of lithium atoms: 1
- cell vectors A x B x C: 36.8927 x 38.3400 x 30.000Å

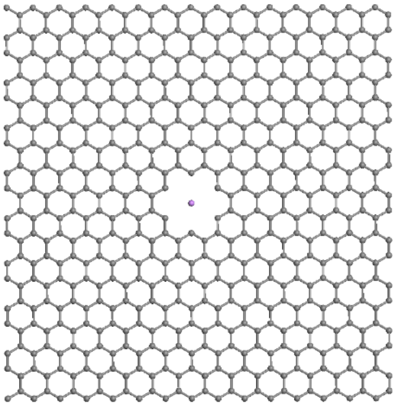

(d), (e) **Graphene nanopore models C24H (24 carbon atoms removed) and C54H (54 carbon atoms removed)**: the C24H and C54H graphene nanopore structures (Figures_2,3 and 4 in the manuscript) were created similarly by removing carbon atoms from the pristine graphene model described above in (a) with the number of carbon atoms in the supercell 516 and 486, respectively

- number of carbon atoms: 516 - for C24H and 486 - for C54H
- number of lithium atoms: 1
- cell vectors A x B x C: 36.8927 x 38.3400 x 30.000Å

C24H: 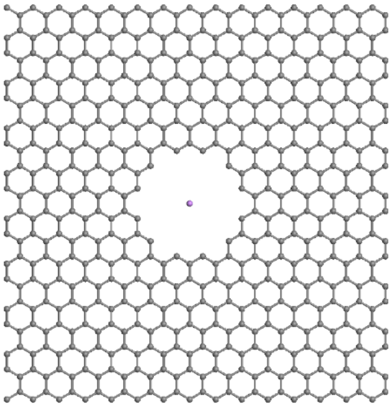 C54H: 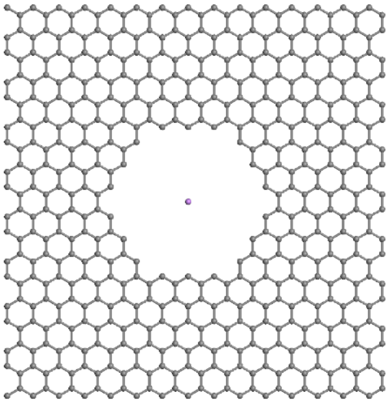

The lithium atom displacement in C6H, C24H and C54H structures was shown in Figure 2 in the manuscript. The calculations were performed for different Li-graphene pore edge separation d_{Li} (Figure 3 in the manuscript) when lithium atom was in plane with graphene nanopore (at z-axis = 0.0nm) and when lithium atom was elevated above the graphene nanopore at (at z-axis = 0.1.72nm). The height of 0.172 nm was chosen since it was found in previous study to be the optimized height of a lithium ion on the pristine graphene.

(f) **Graphene nanopore models C24H and C54H with lithium atoms placement C10, C03, C13, C06, C16:** these structures are based on the C24H and C54H graphene nanopore structures described in (d) and (e) above, but with lithium decorated nanopores that contain more than one lithium atom. The structure descriptions are shown below:

C24H_C10:<br>• number of carbon atoms: 516<br>• number of lithium atoms: 1<br>C24H_C03:<br>• number of carbon atoms: 516<br>• number of lithium atoms: 3<br>C24H_C13:<br>• number of carbon atoms: 516<br>• number of lithium atoms: 4<br>C24H_C06:<br>• number of carbon atoms: 516<br>• number of lithium atoms: 6<br>C24H_C16:<br>• number of carbon atoms: 516<br>• number of lithium atoms: 7	C54H_C10:<br>• number of carbon atoms: 486<br>• number of lithium atoms: 1<br>C54H_C03:<br>• number of carbon atoms: 486<br>• number of lithium atoms: 3<br>C54H_C13:<br>• number of carbon atoms: 486<br>• number of lithium atoms: 4<br>C54H_C06:<br>• number of carbon atoms: 486<br>• number of lithium atoms: 6<br>C54H_C16:<br>• number of carbon atoms: 486<br>• number of lithium atoms: 7

The structures shown in the table above were used to calculate adsorption energy per lithium and per carbon (Figure 5 in the manuscript). The structures with Li atoms at 0.0 nm above the nanopore have all lithium atoms located in graphene plane.

The structures with Li atoms at z-axis distance of 0.172 nm above nanopore have all Li atoms lifted above graphene surface, except for C13 and C16 models (these models have centered lithium atom placed at 0.0 nm and the surrounding Li atoms at 0.172 nm above the graphene plane). The model's illustration is shown below:

C24H_C10 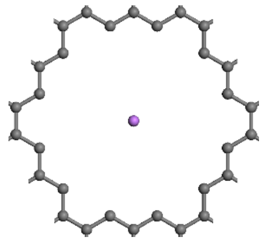	C54H_C10 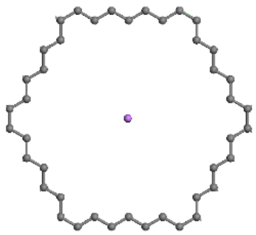	Li atom at 0.0nm above nanopore 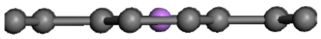	Li atom at 0.172nm above nanopore 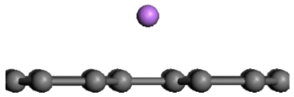
C03 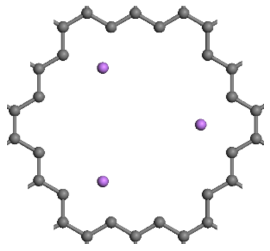	C03 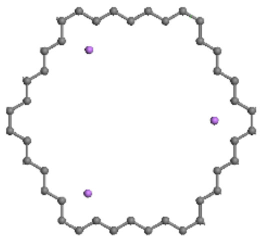	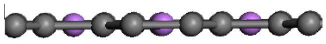	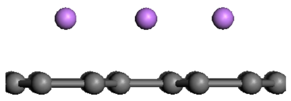
C13	C13	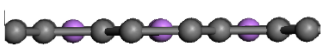	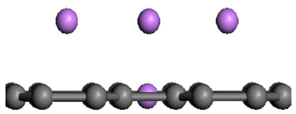

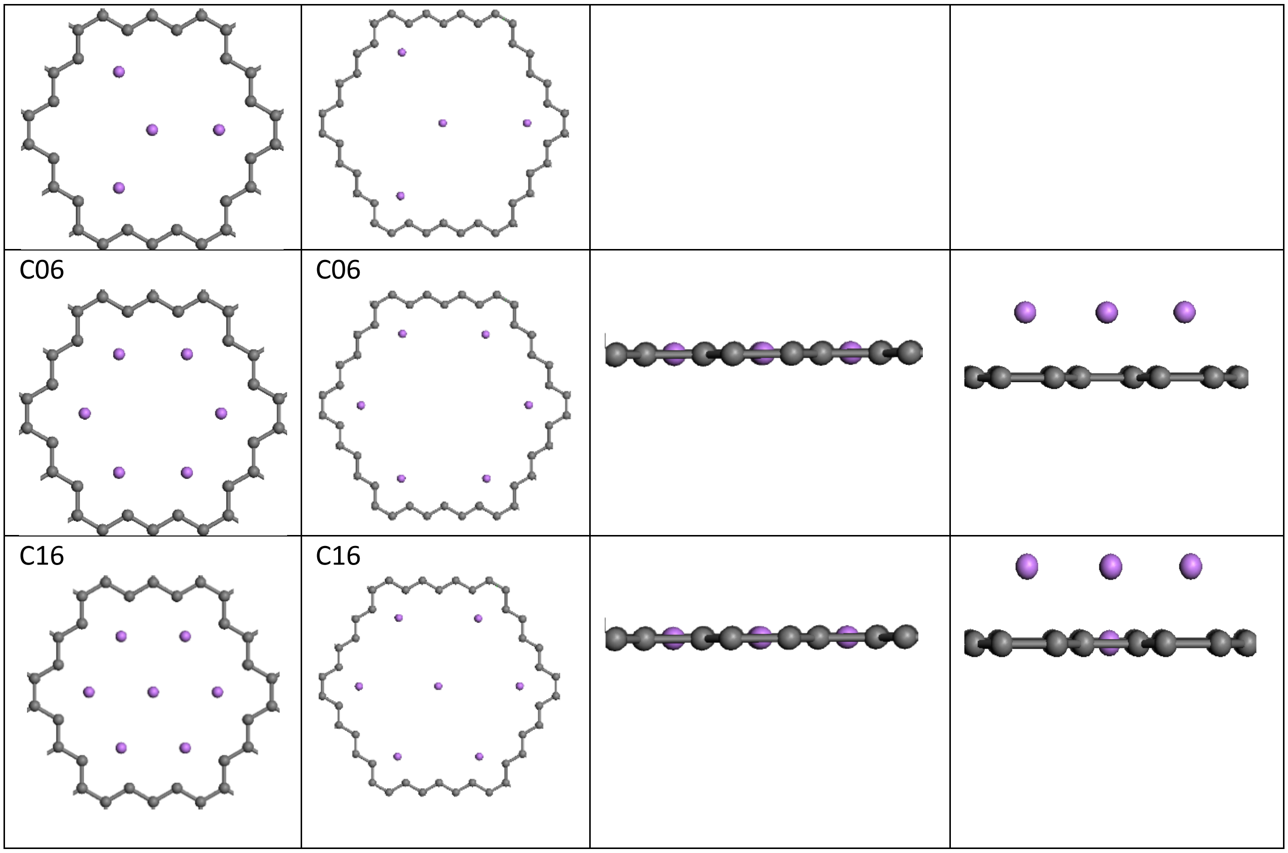

For all models graphene supercell was placed in the box with periodic boundary conditions with cell vectors A x B x C: 36.8927 x 38.3400 x 30.000Å. The graphene sheet was placed at 0.0 Å (0.0nm) and Li atoms was placed in plane with graphene at 0.0Å (0.0nm) and 1.72Å (0.172nm) above the graphene surface.

Figures 6 and 7 in the manuscript have nanoporous graphene structures based on the single layer of pristine graphene nanosheet similarly to the structures from Figures 1-5 above with C54H nanopore size.

Figure 6 contains models 1-4 (models 1,2 – nanopore interior is not Li-decorated; models 3,4 – nanopore interior is decorated with 6 Li atoms).

The detailed description of the models 1-4 are as follows (C-Li and Li-Li separation distances were obtained from the previous study – reference 29):

Model 1(without li atoms in the pore):

- number of carbon atoms: 486
- number of lithium atoms: 209
- G - Li (1st layer) separation 0.184nm
- Li - Li (2nd layer) separation 0.203nm

Model 2 (without li atoms in the pore):

- number of carbon atoms: 486
- number of lithium atoms: 418

- G - Li (1st layer) separation 0.184nm
- Li - Li (2nd layer) separation 0.203nm

Model 3 (with 6 li atoms in the pore at z-axis = 0.0nm):

- number of carbon atoms: 486
- number of lithium atoms: 215
- G - Li (1st layer) separation 0.184nm
- Li - Li (2nd layer) separation 0.203nm

Model 4 (with 6 li atoms in the pore at z-axis = 0.0nm):

- number of carbon atoms: 486
- number of lithium atoms: 424
- G- Li (1st layer) separation 0.184nm
- Li - Li (2nd layer) separation 0.203nm

Figure 7 in the manuscript contains models 5 and 6. Model 5 has bilayer graphene (top layer - C54H nanopore decorated with 6 Li atoms with the Li height of 0.0 nm; the bottom layer is pristine graphene). The lithium atoms from the 1st layer at the height of 0.184nm were eliminated from above and below graphene nanopores decorated with 6 Li atoms to minimize the energy and to get the realistic structure. Model 6 contains a stack of two C54H graphene nanopores without Li atoms in the nanopores. The detailed description of the models 5 and 6 are as follows:

Model 5:

- number of carbon atoms: 1026
- number of lithium atoms: 670
- G - Li (1st layer) separation 0.184nm
- Li - Li (2nd layer) separation 0.203nm
- G - G separation 0.590nm*

Model 6:

- number of carbon atoms: 972
- number of lithium atoms: 418
- G - Li (1st layer) separation 0.184nm
- Li - Li (2nd layer) separation 0.203nm
- G - G separation 0.590nm*

* The energy minimization calculation was performed to determine the separation distance with minimal total potential energy between the graphene layers.

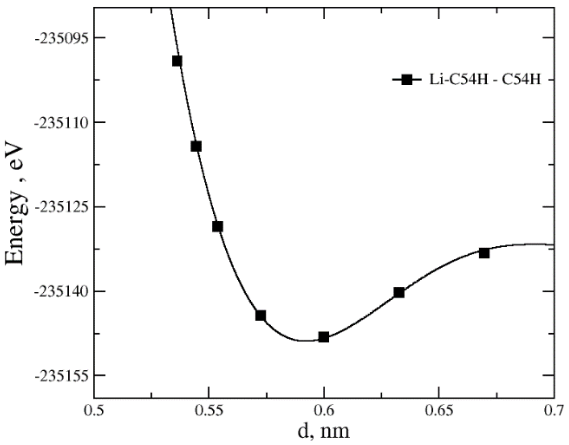

The figure above shows the total potential energy of the whole structure as a function of the separation distance between graphene layers in Models 5-10. For both structures the lowest energy was observed around 0.6nm of the separation distance between the graphene layers and the distance between the bottom graphene supercell and the lithium layer above it was 0.213nm.

Model 10 has higher energy per lithium and energy per carbon than Model 9. The x fraction in Li_xC is 0.798 (closer to 1) for M10 while for M9 this fraction is 0.657:

Model 9:

- number of carbon atoms: 972
- number of lithium atoms: 639
- G - Li (1st layer) separation 0.184nm
- Li - Li (2nd layer) separation 0.203nm
- G-G separation 0.590nm*

Model 10:

- number of carbon atoms: 756
- number of lithium atoms: 603
- G - Li (1st layer) separation 0.184nm
- Li - Li (2nd layer) separation 0.203nm
- G-G separation 0.590nm*
